# Intramuscular Adipose Tissue and the Functional Components of Sarcopenia in Hospitalized Geriatric Patients

**DOI:** 10.3390/geriatrics2010011

**Published:** 2017-02-22

**Authors:** Stany Perkisas, Anne-Marie De Cock, Veronique Verhoeven, Maurits Vandewoude

**Affiliations:** 1 University Center for Geriatrics, ZNA (ZiekenhuisNetwerk Antwerpen), Antwerpen 2000, Belgium; maurits.vandewoude@zna.be; 2 Department of Geriatric Medicine, General Hospital St Maarten, Mechelen 2800, Belgium; Annemarie.DeCock@emmaus.be; 3 ELIZA, First Line & Interdisciplinary Care Medicine, University of Antwerp, Edegem 2650, Belgium; veronique.verhoeven@uantwerpen.be

**Keywords:** sarcopenia, intramuscular adipose tissue, assessment, mortality

## Abstract

Intramuscular adipose tissue (IMAT) could be an important missing value in the assessment of sarcopenia. This study tries to determine the relation between IMAT, muscle strength, functionality and mortality. In addition, the relation with nutritional status is screened. For six months, all patients admitted to the University Geriatric Center of Antwerp were evaluated for strength (hand grip), functionality (short physical performance battery—SPPB) and nutritional status. After one year, patients/relatives were contacted to obtain a current health status (mortality). A total of 303 patients were included at a mean age of 83.0 ± 6.4 years. The mean percentage of IMAT was 29.2% ± 13.0% (range 3.2%–86.2%). There was a negative correlation between IMAT and both grip strength and SPPB. SPPB was positively correlated with both grip strength and muscle mass. There was a positive correlation between IMAT and mortality. There was a negative correlation between grip strength, SPPB and mortality. IMAT did not have a clear relation with nutritional status. IMAT should be addressed in the work-up of sarcopenia, as it is correlated with muscle strength, functionality and mortality. In this cohort of hospitalized geriatric patients, there is a mean of about one-third of measured muscle volume that appears to be adipose tissue.

## 1. Introduction

In recent years, several working groups such as the European Working Group on Sarcopenia in older people, or the Society for Sarcopenia, Cachexia and Wasting disorders, have tried to define an operational concept of sarcopenia [1,2]. The trias muscle mass, muscle strength and muscle performance remain key herein. Some questions remain regarding the place of nutritional state—of which body composition is a part—and, herein, it is very clearly linked with sarcopenia [3,4].

Although there is an existing relationship between muscle mass and muscle strength/performance, it does not follow a linear pattern. This is due to many reasons, both muscular and neurological [5], and gives way to a new meaning of the concept ‘muscle quality’. Whereas it used to be synonymous to force per unit of muscle mass [6,7], muscle quality can now be better defined as meaning muscle composition [8]. One very important feature of muscle composition in the ageing muscle is myosteatosis, which is the increase of lipid depositions in muscle tissue [9]. The processes behind this are still incompletely understood, although it could partly be explained by inadequate function and capacity of subcutaneous depots to store excess fat, increased fatty acid transport, uptake and storage, reduced fatty acid oxidation, increased macrophage infiltration that inhibits adipocyte differentiation, disuse, altered leptin signaling and sex steroid deficiency [10,11]. In addition, there is the theory of an age-related altered differentiation of satellite cells, which are regarded as the muscle stem cells, into adipocytes rather than myocytes [12]. Distinction must be made between steatosis within the myocytes (intramyocellular or intramuscular lipids) and the deposition of lipids in between the myocytes (extramyocellular or intermuscular lipids) [13]. This difference can be made with both computed tomography (CT) and magnetic resonance imaging (MRI) [14], but not with dual-energy X-ray absorptiometry, which, in this account, tends to overestimate the total muscle mass [15,16]. Intramuscular adipose tissue (IMAT) thus mistakenly considered as muscle mass can represent an important bias in the evaluation of the muscle mass component in sarcopenia [17].

Besides having a direct effect on the contractile properties of the muscle bulk, the increased lipid deposition also could have an effect on the central activation of muscles [18] and is known to enhance the local inflammatory pathways in an autocrine/paracrine manner [19]. This chronic inflammation fuels the process of protein catabolism [20] and is thus one of the driving forces behind the process of sarcopenia. Earlier reports have already reported increases in these intramuscular adipose depositions in well-functioning older people, indicating that it is probably part of normal ageing [21,22]. However, certain negative outcomes have been linked to myosteatosis. It is a risk factor for insulin resistance and type 2 diabetes, increased risk of osteoporotic fractures and loss of mobility, decreased muscle strength, reduced physical performance and impaired longevity [10,21,22]. In addition, the link between myosteatosis and mortality is known both in community-dwelling elderly [10,23,24] and in specific populations such as cirrhosis [25] or cancer patients [26,27,28,29]. Nutritional state is not clearly proven to be linked with myosteatosis, unless focusing on animal studies [30].

To our knowledge, no studies have been published about the evaluation of myosteatosis and the relation with the components of sarcopenia and nutrition in an elderly hospitalized population.

## 2. Materials and Methods

### 2.1. Patients

All patients admitted to the University Geriatrics Department from the 1st of August 2012 untill the 31th of January 2013 were screened for nutritional status, muscle mass, muscle strength and functional capacity. There were no exclusion criteria other than the patient refusing the screening tests. After one year, patients or their relatives were contacted by telephone in order to obtain the current health status (mortality).

### 2.2. Measurements

Muscle mass was measured by CT scan (Siemens Somatom Balance, Siemens, Erlangen, Germany) of both upper legs. The midthigh was set by a cranial-caudal scan of the femur where the middle was located half way between the most proximal part of the femur head and the intercondylary region of the distal femur. Five CT-slices, each 1 centimeter apart, were taken above and five slices below this mid-point. The difference between fat, bone, muscle and other tissue could be made by measuring the difference in Hounsfield units. The muscle volume was measured in cubic millimeters (mm^3^) by integration between the slices. Then, the actual muscle weight was calculated in grams (g) by multiplying the volume in mm^3^ by 1.055 g/mm^3^, the assumed constant density of skeletal muscle [31]. In addition, the intra- and extramuscular amount of adipose tissue was measured. This is done as previously described in the literature [32]. The total IMAT is expressed as a percentage of the total measured muscle volume (containing both muscle and IMAT).

Muscle strength was obtained by measuring the hand grip strength with a Jamar dynamometer (Lafayette Instrument, Lafayette, IN, USA). The standard sitting position was used, shoulders were put in a neutral position, and elbows were flexed at a 90° angle. The width of the hand grip of the dynamometer was adjusted to optimally fit in each patient’s hand. Three measurements were done with each hand [33]. In the statistical analysis, the best results of both hands were used. A comparison is made with normative data, which was extracted from a population of 638 healthy volunteers aged 20–94 years old [34].

The functional capacity was measured by performing the Short Physical Performance Battery (SPPB). The SPPB consists of three timed tests: a balance test, a repeated chair stand test and a walking speed test. Each subtest can yield a maximum score of 4, with the maximum total score being 12. Three different groups were considered: those with low (0–4), middle (5–7) and high (8–12) scores, as used by Ostir et al. [35].

Nutritional risk status was measured by both a questionnaire and laboratory parameters. The questionnaire used was the Mini-Nutritional Assessment-Short Form (MNA-SF) [36], taken on the first working day after the day of admission. Patients were classified as: ‘malnourished’ (scores 0–7), ‘at risk of malnourishment’ (scores 8–11), or ‘normal nutritional status’ (scores 12–14). Albumin (normal range 35.0–50.0 g/L), pre-albumin (normal range 17.6–36.0 mg/dL), transferrin (normal range 180–380 mg/dL), C-reactive protein (CRP) (normal range 0.0–5.0 mg/L) and total lymphocyte count (TLC) were measured as nutritional and inflammatory parameters [37].

Comorbidities registered were the presence of diabetes mellitus and conditions leading to immobilization, such as a cerebrovascular accident, a fracture of the vertebrae, the femur or the humerus, any type of surgery (orthopedic or otherwise), either planned or in an emergency setting, and staying in an intensive care unit.

### 2.3. Statistics

Statistical analysis was done with SPSS 20 (SPSS Inc., Chicago, IL, USA). Descriptive statistics describe demographic and key clinical characteristics of the study population. A Kolmogorov–Smirnov test was used for assumption of normality. Student’s *t*-tests were used to test for differences in the distribution of continuous variables. A chi-square test was used for significance of associations with categorical variables. *p*-values < 0.05 were considered statistically significant. *p*-values were adjusted for multiple comparisons using the Bonferroni method. Correlations were measured by Pearson’s correlation coefficient (PCC).

### 2.4. Informed Consent

Oral informed consent was obtained from all patients regarding blood analysis, mini-mental state examination (MMSE), handgrip measurement and SPPB. Sixty patients did not give their informed consent for muscle mass measurements with a CT-scan.

## 3. Results

### 3.1. Baseline Characteristics

Of the 303 included patients, 211 (69.6%) were women. The mean age was 83.0 ± 6.4 years (range 64–101 years). Further baseline characteristics are summarized in Table 1. One hundred percent follow-up was obtained. Mean follow-up time after admittance was 344 ± 127 days (range 2–530 days). Seventy-six patients died during the period of follow-up.

### 3.2. Muscle Mass

Radiologic data were collected from 199 patients (65.7%). Of these, 140 (70.4%) were women. As some hip implants (long-stemmed) give too much distortion for correct measurements, 44 patients did not undergo a CT scan. Sixty patients did not give their informed consent for muscle mass measurements with a CT-scan.

Values of muscle mass and intramuscular adipose tissue are given in Table 2. There was no significant difference in values between the left and right side. Men had more muscle mass (*p* < 0.001) and less intramuscular fat tissue (*p* < 0.001) than women. There was a relative high mean percentage of intramuscular adipose tissue in both groups (29.3% for the left leg, 29.1% for the right leg) with a large range (3.2%–86.2%). The IMAT did not have a relevant correlation with length of stay (LOS). There was a positive correlation with mortality (*p* = 0.014, PCC −0.174).

### 3.3. Muscle Strength

Overall mean handgrip strength of the left hand was 12.6 ± 8.6 kg, with a median of 12.0 kg (range 0–48 kg). Mean handgrip strength of the right hand was 13.7 ± 9.1 kg, and the median was 12.0 kg (range 0–52 kg). The difference between the handgrip strength of the left and right hands was significant (*p* = 0.001). Mean handgrip strength in men (strongest hand) was 16.6 ± 9.2 kg, and the median was 16.0 kg (range 0–43 kg). Mean handgrip strength in women (strongest hand) was 14.4 ± 8.8 kg, and the median was 14.0 kg (range 0–52 kg). The difference between the handgrip strength of men and women was significant (*p* < 0.001). Handgrip strength according to age group compared to normative data is given in Table 3. In this sample of hospitalized patients, hand grip strength was significantly lower than those given by the normative data. There was a clear negative correlation between hand grip strength and IMAT. This was true for every measurement, whether the left hand (*p* = 0.001, PCC −0.251), the right hand (*p* = 0.001, PCC −0.331) or the best measurement of both hands was selected (*p* < 0.001, PCC −0.323). Handgrip strength of men was not correlated with IMAT (*p* = 0.126) but that of women was (*p* < 0.001). Handgrip strength did not have a relevant correlation with a length of stay (LOS). There was a negative correlation with mortality (*p* = 0.014, PCC 0.149).

### 3.4. Functionality

Mean SPPB-score was 4 ± 3, and the median was 3 (range 0–12). SPPB-scores were negatively correlated with CRP (*p* = 0.032, PCC −0.121) and positively correlated with both handgrip strength (*p* < 0.001, PCC 0.311) and muscle mass (*p* < 0.001, PCC 0.406). There was a negative correlation between SPPB-scores and IMAT in this population (*p* < 0.001, PCC −0.450). SPPB did not have a relevant correlation with length of stay (LOS). There was a negative correlation with mortality (*p* = 0.001, PCC 0.196).

### 3.5. Nutritional Risk

The mean score on the MNA-SF was 9.2 ± 3.2, and the median value was 9.0 (range 0–14). There were 88 patients (29.0%) classified in the ‘malnourished’ group, 127 patients (42.0%) in the ‘at risk of malnourishment’ group and 88 patients (29.0%) in the ‘normal nutritional status’ group. For the distribution of the laboratory values, we refer to Table 1. Only in the cases of albumin and pre-albumin could the null hypothesis not be rejected. Higher scores on MNA-SF were correlated with better SPPB-scores (*p* = 0.006, PCC 0.232), higher handgrip strength (*p* < 0.001, PCC 0.102), and higher muscle mass (*p* < 0.001, PCC 0.319). There was no clear relation between MNA-SF and IMAT (*p* = 0.328). Nutritional status did not have a relevant correlation with length of stay (LOS). There was a negative correlation with mortality (*p* < 0.001, PCC 0.303).

Of the laboratory parameters, only CRP was significant related to SPPB-scores (*p* = 0.032, PCC −0.121). There was a positive correlation between hand grip strength and both albumin (*p* < 0.001, PCC 0.134) and TLC (*p* < 0.001, PCC 0.143).

### 3.6. Other

Recent events with a possible effect on mobility, and thus on strength/functionality, were noted. Among these were vertebral fractures (*n* = 21, 6.9%), pelvic fractures (*n* = 12, 4.0%), fractures of either the lower limb (*n* = 46, 15.2%) or upper limb (*n* = 22, 7.3%), CVA with hemiplegia (*n* = 19, 6.3%), elective surgery on the lower limb (*n* = 13, 4.3%), oncologic surgery (*n* = 12, 4.0%), other types of surgery (*n* = 1, 0.3%), and stay in the intensive care ward (*n* = 10, 3.3%). In 147 patients (48.4%), there was no specific reason for immobilization at all.

## 4. Discussion

To our knowledge, this is the first study to examine the relationship between IMAT and parameters of sarcopenia and nutrition in hospitalized geriatric patients. Due to a large heterogeneity in older people, hospitalized geriatric patients differ significantly from relatively healthy community-dwelling elderly. This is, for example, shown by the fact that mean handgrip measurements in our sample were much lower than known normative data, even when corrected for age.

In this cohort, IMAT was negatively correlated with muscle strength and functionality, and positively correlated with one-year mortality. This data in hospitalized patients strengthens the evidence already known in other cohorts. Seeing that there is an important correlation with mortality, and also that in this specific population there is a very large range of IMAT when measuring muscle mass, it is seen as paramount that, when assessing sarcopenia, a measurement of IMAT must be done.

Our methodology for muscle mass determination differs from other study groups in literature, as we preferred to integrate the muscle volume measured by CT over a distance of 10 cm instead of 1 cm. This levels out the significant variability of distribution of muscle and adipose tissue in different parts of the thigh. Because the muscles of the upper leg are essential for activities of daily living and mobility (standing up, walking around, climbing stairs), it seems mere logic to take them as reference.

**Figure 1:**
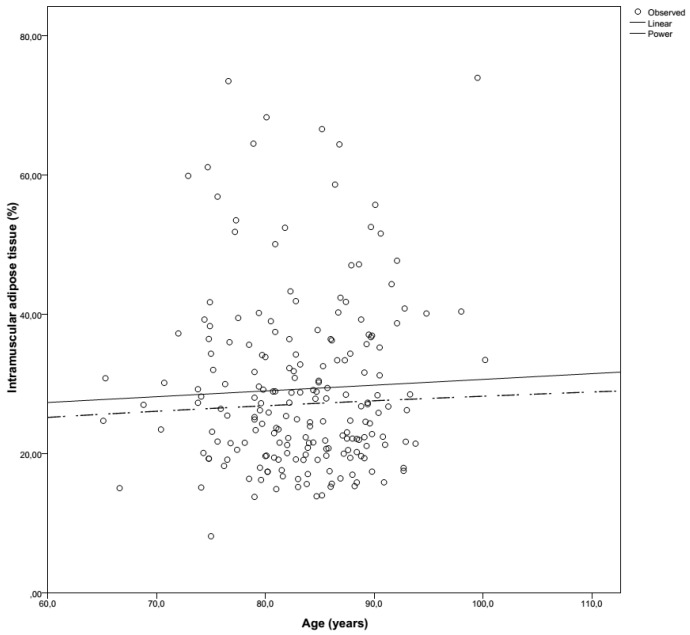
Intramuscular adipose tissue (%) evolution according to age.

**Figure 2:**
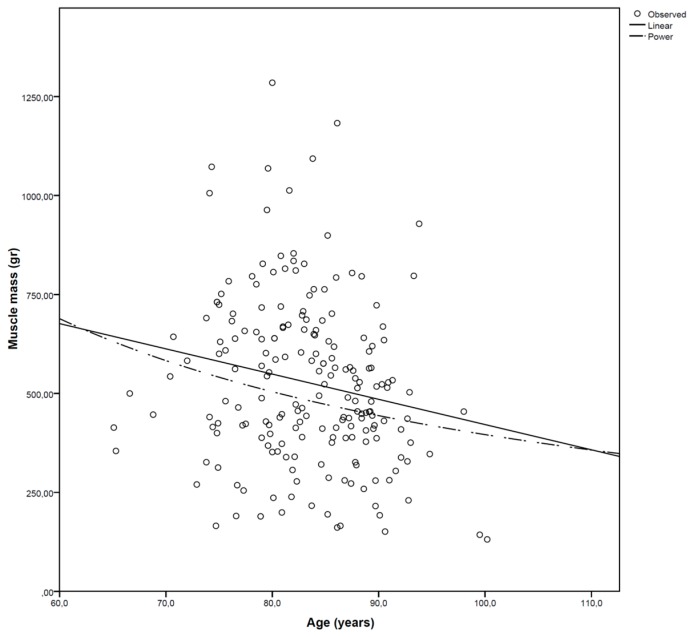
Muscle mass (gram) evolution according to age.

The amount of adipose tissue in muscle in this hospitalized cohort was also much higher than previously described in the literature. Goodpaster et al. used CT imaging to make a 10-mm tick slice of the upper leg in healthy older adults, in whom there was an IMAT percentage of 4.8% and 5.1% [38]. Delmonico et al. measured the muscle adipose tissue longitudinally in ‘young old’ healthy community-dwelling people for five years [22]. At baseline, white men had an IMAT percentage of 6.7%, where black men, white women and black women had IMAT percentages of 7%, 9% and 10.9%, respectively. In our cohort of hospitalized patients, a much higher mean intramuscular adipose tissue content (±29%) was found. This was a consistent finding in both genders and both legs (Table 2). Although there is a progressive increase in muscle adipose tissue content with ageing (Figure 1), the rate of fat accretion is not equal to the loss of muscle (Figure 2). The exact rate of this ‘transformation’ in acutely ill patients is unclear.

It is known that older people lose a significant amount of muscle when they are immobilized [39]. We note a progressive decline of muscle mass and an increase in IMAT with longer hospitalization; it must be noted, however, that correlation does not prove causality. The mechanism is not clear, but in vitro studies point towards the pluripotent capacity of progenitor cells of myocytes who can differentiate into adipocytes in response to various stimuli [12].

At first, it was thought that the loss of muscle mass was the reason for loss of strength and functionality. Nowadays, we know that this relation is much more complex [40]. There seem to be both muscular and neural factors that contribute to overall muscle weakness [41]. A major determinant of this weakness might be the presence of IMAT. Because adipose tissue infiltration in the muscle can be very substantial, and in extreme cases rises above 80%, this can be an important factor in the non-linear relationship between muscle mass and functionality. In our sample, a clear inverse relationship between IMAT and both strength and functionality was noted, supporting this hypothesis.

**Figure 3:**
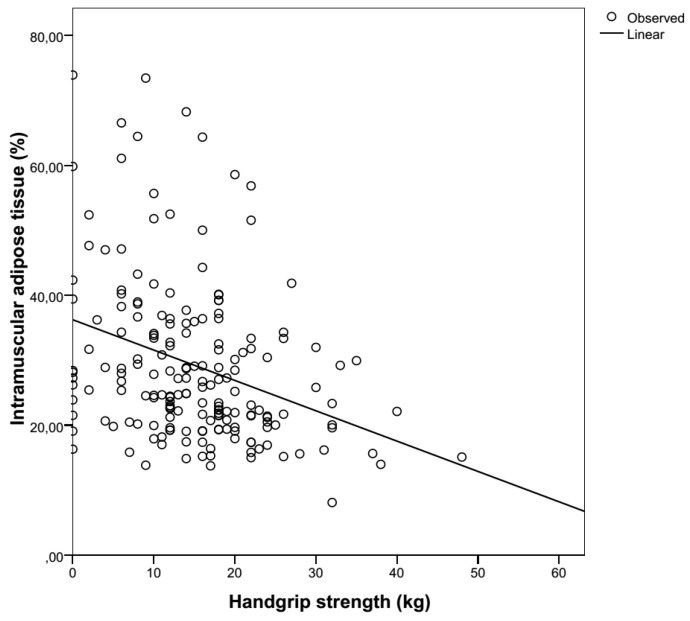
The inverse relationship between intramuscular adipose tissue (%) and handgrip strength (kg).

Hand grip strength is known to be negatively correlated with length of hospitalization, rehospitalization rate and mortality, and positively correlated with functional status [42,43,44]. These findings fit those of our population, where handgrip strength was correlated positively with functionality and negatively with one-year mortality. It comes as no surprise that it is also negatively correlated with IMAT (Figure 3), as seen in the literature [45,46]. In this cohort, there is a positive correlation with nutritional state (MNA-SF), which is also seen in other studies [47].

**Figure 4:**
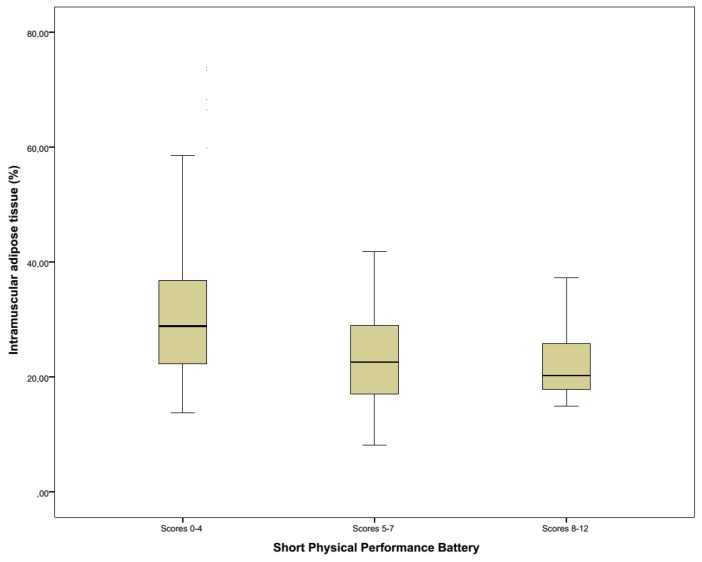
Relationship between intramuscular adipose tissue (%) and functionality according to the Short Physical Performance Battery (divided in low, mediocre and high scores).

The SPPB is one of the most validated and used performance tests, specifically for the lower extremity function, which is most relevant for day-to-day tasks, e.g., walking or standing up. It is a good predictor for long term disability and institutionalization [48,49], and the different components of the SPPB are all associated with mortality [50]. In this cohort, SPPB positively correlates with nutritional status (MNA-SF) and handgrip strength. A strong negative correlation was seen between the SPPB and IMAT in this population (Figure 4), and also with mortality. This is consistent with the current literature [51].

Malnutrition was very common in this hospitalized cohort. Seventy-one percent was either malnourished or at a risk of malnourishment, which is significantly higher than in other settings such as the community or nursing homes [52]. This high rate of malnutrition was consistent with the laboratory work-up, where 76.2% (*n* = 231) had albumin levels below normal and 37.0% (*n* = 121) had low pre-albumin levels. This is important because an adequate intake of proteins is a key component in the prevention of sarcopenia, and treatment of protein malnutrition can improve muscle mass and strength [53]. Our data are consistent with the current literature [47,54], showing a correlation between (risk of) malnutrition and lower muscle strength and performance, strengthening the concept of the malnutrition–sarcopenia syndrome [55]. There is also a clear link between malnutrition and mortality in this cohort.

There are a few limitations to the study. Although an extended list was used, not all comorbidities were taken into account. In addition, many patients were referred for revalidation after stroke or orthopedic surgery, which might have had an effect on overall muscle mass or functionality. However, this pathology is typical for geriatric and rehabilitation wards. Conclusions, therefore, should be restricted to this type of patient and not to the well-functioning community-dwelling elderly. The strengths of this study are the large sample of hospitalized patients and the complete follow-up data.

## 5. Conclusions

In conclusion, IMAT should be addressed in the work-up of sarcopenia, as it is correlated with muscle strength, functionality and mortality. On average, about one-third of the muscle volume measured in hospital-admitted geriatric patients turns out to be adipose tissue. This will strongly interfere with assessment techniques of muscle mass and can lead to difficult and perhaps faulty interpretation of these results. Therefore, clinicians are urged to screen for and assess this specific parameter of sarcopenia, which is, at present, not yet taken into account in standard screening protocols.

## Figures and Tables

**Table 1:** Baseline characteristics of hospitalized geriatric patients (*n* = 303).

Parameters	Mean	Median	Range
Patient characteristics			
Age (years)	83.0 ± 6.4	83	64–101
Length (cm)	163 ± 8	163	142–186
Weight (kg)	65.9 ± 15.4	64.0	33.2–154.5
BMI (kg/m^2^)	24.7 ± 5.3	23.9	14.2–47.4
Laboratory			
Pre-albumin (mg/dL)	20.0 ± 7.1	20.0	5–43
Albumin (g/L)	31.3 ± 5.5	31.0	11–53
CRP (mg/dL)	2.6 ± 3.6	1.0	0–22
Vitamin D (ng/dL)	17.5 ± 12.2	14.0	3–69
Transferrin (mg/dL)	195 ± 47	190	91–384
Total lymphocyte count	1.63 ± 0.93	2.05	0–9

Cm = centimeter, kg = kilogram, m = meter, mg = milligram, dL = deciliter, g = gram, L = liter, ng = nanogram. BMI = body mass index.

**Table 2:** Muscle mass (gr) and intramuscular adipose tissue (IMAT, %) values in men and women from both left and right legs.

Measurements	Mean		*p*-Value	Median		Range	
	Left	Right		Left	Right	Left	Right
Overall muscle mass	522 ± 15	533 ± 16	0.080	484	518	114–1244	60–1325
Men	549 ± 215	565 ± 226	0.213	548	545	123–1197	97–1169
Women	511 ± 209	518 ± 216	0.213	470	472	114–1244	60–1325
Overall IMAT (%)	29.3 ± 13.4	29.1 ± 12.5	0.751	25.8	25.6	3.2–82.7	8.5–86.2
Men	27.2 ± 11.0	26.9 ± 11.9	0.731	23.8	22.9	13.5–56.7	14.0–63.0
Women	30.2 ± 14.3	30.0 ± 12.6	0.860	26.9	26.6	3.2–82.7	8.5–86.2

**Table 3:** Handgrip strength: normative data from Mathiowetz et al. [34] compared to own data.

			Male		Female	
			Normative Data	Current Data	Normative Data	Current Data
Age	N	Hand	Mean ± SD (kg)	Mean ± SD (kg)	Mean ± SD (kg)	Mean ± SD (kg)
65–69	3	R	41.3 ± 9.3	0.0 ± NA	22.5 ± 4.4	5.5 ± 7.8
		L	34.8 ± 9.0	22.0 ± 22.0	18.6 ± 3.7	4.0 ± 5.7
70–74	24	R	34.2 ± 9.8	14.3 ± 16.3	22.5 ± 5.3	16.4 ± 9.8
		L	29.4 ± 8.2	10.3 ± 10.0	18.8 ± 4.6	13.0 ± 9.2
75+	246	R	29.8 ± 9.5	14.7 ± 9.2	19.3 ± 5.0	13.0 ± 8.8
		L	24.9 ± 7.7	14.0 ± 8.9	17.1 ± 4.0	11.9 ± 8.3

## References

[1] Cruz-Jentoft A.J., Baeyens J.P., Bauer J.M., Boirie Y., Cederholm T., Landi F., Martin F.C., Michel J.P., Rolland Y., Schneider S.M. (2010). Sarcopenia: European consensus on definition and diagnosis: Report of the European Working Group on Sarcopenia in Older People. Age Ageing.

[2] Morley J.E., Abbatecola A.M., Argiles J.M., Baracos V., Bauer J., Bhasin S., Cederholm T., Coats A.J., Cummings S.R., Evans W.J. (2011). Sarcopenia with limited mobility: An international consensus. J. Am. Med. Dir. Assoc..

[3] Komai S., Watanabe Y., Fujiwara Y., Kim H., Edahiro A., Kawai H., Yoshida H., Obuchi S., Tanaka Y., Hirano H. (2016). Association between the nutritional status and the severity of sarcopenia among community-dwelling elderly Japanese people. Nihon Ronen Igakkai zasshi Jpn. J. Geriatr..

[4] Lancha A.H., Zanella R., Tanabe S.G., Andriamihaja M., Blachier F. (2017). Dietary protein supplementation in the elderly for limiting muscle mass loss. Amino Acids.

[5] Perkisas S., De Cock A., Verhoeven V., Vandewoude M. (2016). Physiological and architectural changes in the ageing muscle and their relation to strength and function in sarcopenia. Eur. Geriatr. Med..

[6] Metter E.J., Lynch N., Conwit R., Lindle R., Tobin J., Hurley B. (1999). Muscle quality and age: Cross-sectional and longitudinal comparisons. J. Gerontol. Ser. A.

[7] Narici M.V., Reeves N.D., Morse C.I., Maganaris C.N. (2004). Muscular adaptations to resistance exercise in the elderly. J. Musculoskelet. Neuronal Interact..

[8] Heymsfield S.B., Gonzalez M.C., Lu J., Jia G., Zheng J. (2015). Skeletal muscle mass and quality: Evolution of modern measurement concepts in the context of sarcopenia. Proc. Nutr. Soc..

[9] Kuk J.L., Saunders T.J., Davidson L.E., Ross R. (2009). Age-related changes in total and regional fat distribution. Ageing Res. Rev..

[10] Miljkovic I., Kuipers A.L., Cauley J.A., Prasad T., Lee C.G., Ensrud K.E., Cawthon P.M., Hoffman A.R., Dam T.T., Gordon C.L. (2015). Greater Skeletal Muscle Fat Infiltration Is Associated With Higher All-Cause and Cardiovascular Mortality in Older Men. J. Gerontol. Ser. A.

[11] Hamrick M.W., McGee-Lawrence M.E., Frechette D.M. (2016). Fatty Infiltration of Skeletal Muscle: Mechanisms and Comparisons with Bone Marrow Adiposity. Front. Endocrinol..

[12] Sinanan A.C., Buxton P.G., Lewis M.P. (2006). Muscling in on stem cells. Biol. Cell.

[13] Miljkovic I., Zmuda J.M. (2010). Epidemiology of myosteatosis. Curr. Opin. Clin. Nutr. Metab. Care.

[14] Larson-Meyer D.E., Smith S.R., Heilbronn L.K., Kelley D.E., Ravussin E., Newcomer B.R. (2006). Muscle-associated triglyceride measured by computed tomography and magnetic resonance spectroscopy. Obesity.

[15] Reiner A.P., Aragaki A.K., Gray S.L., Wactawski-Wende J., Cauley J.A., Cochrane B.B., Kooperberg C.L., Woods N.F., LaCroix A.Z. (2009). Inflammation and thrombosis biomarkers and incident frailty in postmenopausal women. Am. J. Med..

[16] Beasley J.M., LaCroix A.Z., Neuhouser M.L., Huang Y., Tinker L., Woods N., Michael Y., Curb J.D., Prentice R.L. (2010). Protein intake and incident frailty in the Women’s Health Initiative observational study. J. Am. Geriatr. Soc..

[17] Levine J.A., Abboud L., Barry M., Reed J.E., Sheedy P.F., Jensen M.D. (2000). Measuring leg muscle and fat mass in humans: Comparison of CT and dual-energy X-ray absorptiometry. J. Appl. Physiol..

[18] Yoshida Y., Marcus R.L., Lastayo P.C. (2012). Intramuscular adipose tissue and central activation in older adults. Muscle Nerve.

[19] Buch A., Carmeli E., Boker L.K., Marcus Y., Shefer G., Kis O., Berner Y., Stern N. (2016). Muscle function and fat content in relation to sarcopenia, obesity and frailty of old age—An overview. Exp. Gerontol..

[20] Guillet C., Masgrau A., Walrand S., Boirie Y. (2012). Impaired protein metabolism: Interlinks between obesity, insulin resistance and inflammation. Obes. Rev..

[21] Tuttle L.J., Sinacore D.R., Mueller M.J. (2012). Intermuscular adipose tissue is muscle specific and associated with poor functional performance. J. Aging Res..

[22] Delmonico M.J., Harris T.B., Visser M., Park S.W., Conroy M.B., Velasquez-Mieyer P., Boudreau R., Manini T.M., Nevitt M., Newman A.B. (2009). Longitudinal study of muscle strength, quality, and adipose tissue infiltration. Am. J. Clin. Nutr..

[23] Reinders I., Murphy R.A., Brouwer I.A., Visser M., Launer L., Siggeirsdottir K., Eiriksdottir G., Gudnason V., Jonsson P.V., Lang T.F. (2016). Muscle Quality and Myosteatosis: Novel Associations With Mortality Risk: The Age, Gene/Environment Susceptibility (AGES)-Reykjavik Study. Am. J. Epidemiol..

[24] Zhao Q., Zmuda J.M., Kuipers A.L., Jonnalagadda P., Bunker C.H., Patrick A.L., Youk A.O., Miljkovic I. (2016). Greater skeletal muscle fat infiltration is associated with higher all-cause mortality among men of African ancestry. Age Ageing.

[25] Montano-Loza A.J., Angulo P., Meza-Junco J., Prado C.M., Sawyer M.B., Beaumont C., Esfandiari N., Ma M., Baracos V.E. (2016). Sarcopenic obesity and myosteatosis are associated with higher mortality in patients with cirrhosis. J. Cachexia Sarcopenia Muscle.

[26] Okumura S., Kaido T., Hamaguchi Y., Kobayashi A., Shirai H., Fujimoto Y., Iida T., Yagi S., Taura K., Hatano E. (2016). Impact of Skeletal Muscle Mass, Muscle Quality, and Visceral Adiposity on Outcomes Following Resection of Intrahepatic Cholangiocarcinoma. Ann. Surg. Oncol..

[27] Malietzis G., Currie A.C., Athanasiou T., Johns N., Anyamene N., Glynne-Jones R., Kennedy R.H., Fearon K.C., Jenkins J.T. (2016). Influence of body composition profile on outcomes following colorectal cancer surgery. Br. J. Surg..

[28] Kaibori M., Ishizaki M., Iida H., Matsui K., Sakaguchi T., Inoue K., Mizuta T., Ide Y., Iwasaka J., Kimura Y. (2015). Effect of Intramuscular Adipose Tissue Content on Prognosis in Patients Undergoing Hepatocellular Carcinoma Resection. J. Gastrointest. Surg..

[29] Kumar A., Moynagh M.R., Multinu F., Cliby W.A., McGree M.E., Weaver A.L., Young P.M., Bakkum-Gamez J.N., Langstraat C.L., Dowdy S.C. (2016). Muscle composition measured by CT scan is a measurable predictor of overall survival in advanced ovarian cancer. Gynecol. Oncol..

[30] Powell D.J., Velleman S.G., Cowieson A.J., Singh M., Muir W.I. (2016). Influence of hatch time and access to feed on intramuscular adipose tissue deposition in broilers. Poultry Sci..

[31] Ward S.R., Lieber R.L. (2005). Density and hydration of fresh and fixed human skeletal muscle. J. Biomech..

[32] Visser M., Kritchevsky S.B., Goodpaster B.H., Newman A.B., Nevitt M., Stamm E., Harris T.B. (2002). Leg muscle mass and composition in relation to lower extremity performance in men and women aged 70 to 79: The health, aging and body composition study. J. Am. Geriatr. Soc..

[33] Roberts H.C., Denison H.J., Martin H.J., Patel H.P., Syddall H., Cooper C., Sayer A.A. (2011). A review of the measurement of grip strength in clinical and epidemiological studies: Towards a standardised approach. Age Ageing.

[34] Mathiowetz V., Kashman N., Volland G., Weber K., Dowe M., Rogers S. (1985). Grip and pinch strength: Normative data for adults. Arch. Phys. Med. Rehabil..

[35] Ostir G.V., Volpato S., Fried L.P., Chaves P., Guralnik J.M. (2002). Reliability and sensitivity to change assessed for a summary measure of lower body function: Results from the Women’s Health and Aging Study. J. Clin. Epidemiol..

[36] Guigoz Y., Vellas B., Garry P.J. (1996). Assessing the nutritional status of the elderly: The Mini Nutritional Assessment as part of the geriatric evaluation. Nutr. Rev..

[37] Omran M.L., Morley J.E. (2000). Assessment of protein energy malnutrition in older persons, Part II: Laboratory evaluation. Nutrition.

[38] Goodpaster B.H., Chomentowski P., Ward B.K., Rossi A., Glynn N.W., Delmonico M.J., Kritchevsky S.B., Pahor M., Newman A.B. (2008). Effects of physical activity on strength and skeletal muscle fat infiltration in older adults: A randomized controlled trial. J. Appl. Physiol..

[39] Kortebein P., Ferrando A., Lombeida J., Wolfe R., Evans W.J. (2007). Effect of 10 days of bed rest on skeletal muscle in healthy older adults. JAMA.

[40] Morley J.E., Thomas D.R., Wilson M.M. (2006). Cachexia: Pathophysiology and clinical relevance. Am. J. Clin. Nutr..

[41] Perkisas S., Vandewoude M. (2016). Where frailty meets diabetes. Diabetes Metab. Res. Rev..

[42] Keevil V., Mazzuin Razali R., Chin A.V., Jameson K., Aihie Sayer A., Roberts H. (2013). Grip strength in a cohort of older medical inpatients in Malaysia: A pilot study to describe the range, determinants and association with length of hospital stay. Arch. Gerontol. Geriatr..

[43] Simmonds S.J., Syddall H.E., Westbury L.D., Dodds R.M., Cooper C., Aihie Sayer A. (2015). Grip strength among community-dwelling older people predicts hospital admission during the following decade. Age Ageing.

[44] Leong D.P., Teo K.K., Rangarajan S., Lopez-Jaramillo P., Avezum A., Orlandini A., Seron P., Ahmed S.H., Rosengren A., Kelishadi R. (2015). Prognostic value of grip strength: Findings from the Prospective Urban Rural Epidemiology (PURE) study. Lancet.

[45] Almurdhi M.M., Reeves N.D., Bowling F.L., Boulton A.J., Jeziorska M., Malik R.A. (2016). Reduced Lower-Limb Muscle Strength and Volume in Patients with Type 2 Diabetes in Relation to Neuropathy, Intramuscular Fat, and Vitamin D Levels. Diabetes Care.

[46] Maly M.R., Calder K.M., Macintyre N.J., Beattie K.A. (2013). Relationship of intermuscular fat volume in the thigh with knee extensor strength and physical performance in women at risk of or with knee osteoarthritis. Arthritis Care Res..

[47] Mithal A., Bonjour J.P., Boonen S., Burckhardt P., Degens H., Hajj E.l., Fuleihan G., Josse R., Lips P., Morales T.J., Rizzoli R. (2013). Impact of nutrition on muscle mass, strength, and performance in older adults. Osteoporos. Int..

[48] Guralnik J.M., Ferrucci L., Pieper C.F., Leveille S.G., Markides K.S., Ostir G.V., Studenski S., Berkman L.F., Wallace R.B. (2000). Lower extremity function and subsequent disability: Consistency across studies, predictive models, and value of gait speed alone compared with the short physical performance battery. J. Gerontol. Ser. A.

[49] Guralnik J.M., Simonsick E.M., Ferrucci L., Glynn R.J., Berkman L.F., Blazer D.G., Scherr P.A., Wallace R.B. (1994). A short physical performance battery assessing lower extremity function: Association with self-reported disability and prediction of mortality and nursing home admission. J. Gerontol..

[50] Cooper R., Kuh D., Hardy R. (2010). Objectively measured physical capability levels and mortality: Systematic review and meta-analysis. BMJ.

[51] Nam S., Al Snih S., Markides K. (2016). Lower body function as a predictor of mortality over 13 years of follow up: Findings from Hispanic Established Population for the Epidemiological Study of the Elderly. Geriatr. Gerontol. Int..

[52] Vandewoude M., Van Gossum A. (2013). Nutritional screening strategy in nonagenarians: The value of the MNA-SF (mini nutritional assessment short form) in NutriAction. J. Nutr. Health Aging..

[53] Denison H.J., Cooper C., Sayer A.A., Robinson S.M. (2015). Prevention and optimal management of sarcopenia: A review of combined exercise and nutrition interventions to improve muscle outcomes in older people. Clin. Interv. Aging.

[54] Volkert D. (2011). The role of nutrition in the prevention of sarcopenia. Wiener Med. Wochenschr..

[55] Vandewoude M.F., Alish C.J., Sauer A.C., Hegazi R.A. (2012). Malnutrition-sarcopenia syndrome: Is this the future of nutrition screening and assessment for older adults?. J. Aging Res..

